# Association of thrombocytopenia and infection in patients with ST-elevation myocardial infarction undergoing percutaneous coronary intervention

**DOI:** 10.1186/s12872-021-02210-3

**Published:** 2021-08-21

**Authors:** Litao Wang, Weijiang Su, Jinhua Xue, Xiao Gong, Yining Dai, Jiyan Chen, Ling Xue, Pengcheng He, Yuanhui Liu, Ning Tan

**Affiliations:** 1 Department of Cardiology, Guangdong Cardiovascular Institute, Guangdong Provincial Key Laboratory of Coronary Heart Disease Prevention, Guangdong Provincial People’s Hospital, Guangdong Academy of Medical Sciences, Guangzhou, 510100 China; 2 Guangdong Provincial People’s Hospital, School of Medicine, South China University of Technology, Guangzhou, 510100 China; 3 Department of Cardiology, The People’s Hospital of Dianbai District, Maoming, 525400 China; 4 Department of Physiology, School of Basic Medical Sciences, Key Laboratory of Prevention and Treatment of Cardiovascular and Cerebrovascular Diseases of Ministry of Education, Gannan Medical University, Ganzhou, 341000 China; 5 School of Public Health, Guangdong Pharmaceutical University, Guangzhou, 510006 China; 6 The Second School of Clinical Medicine, Southern Medical University, Guangzhou, 510515 China

**Keywords:** Thrombocytopenia, Infection, ST-elevation myocardial infarction, Percutaneous coronary intervention

## Abstract

**Background:**

The impact of thrombocytopenia on infection in patients with ST-elevation myocardial infarction (STEMI) remains poorly understood.

**Aims:**

To evaluate the association between thrombocytopenia and infection in patients with STEMI.

**Methods:**

Patients diagnosed with STEMI were identified from January 2010 to June 2016. The primary endpoint was in-hospital infection, and major adverse clinical events (MACE) and all-cause death were considered as secondary endpoints.

**Results:**

A total of 1401 STEMI patients were enrolled and divided into two groups according to the presence (n = 186) or absence (n = 1215) of thrombocytopenia. The prevalence of in-hospital infection was significantly higher in the thrombocytopenic group (30.6% (57/186) vs. 16.2% (197/1215), *p* < 0.001). Prevalence of in-hospital MACE (30.1% (56/186) vs. 16.4% (199/1215), *p* < 0.001) and all-cause death (8.1% (15/186) vs. 3.8% (46/1215), *p* = 0.008) revealed an increasing trend. Multivariate analysis indicated that thrombocytopenia was independently associated with increased in-hospital infection (OR, 2.09; 95%CI 1.32–3.27; *p* = 0.001) and MACE (1.92; 1.27–2.87; *p* = 0.002), but not all-cause death (1.87; 0.88–3.78; *p* = 0.091). After a median follow-up of 2.85 years, thrombocytopenia was not associated with all-cause death at multivariable analysis (adjusted hazard ratio, 1.19; 95%CI 0.80–1.77; *p* = 0.383).

**Conclusions:**

Thrombocytopenia is significantly correlated with in-hospital infection and MACE, and might be used as a prognostic tool in patients with STEMI.

**Supplementary Information:**

The online version contains supplementary material available at 10.1186/s12872-021-02210-3.

## Background

Infection is an uncommon (reported prevalence of 2.4%) but potentially devastating complication in patients with ST-elevation myocardial infarction (STEMI). Infection in these patients is usually associated with a significantly increased risk of mortality and morbidity, prolongs hospitalization, and increases healthcare costs [1–3]. Given most infections are considered preventable, there is an urgent need to identify STEMI patients at high risk of infection, and implement interventions to prevent infection.

Thrombocytopenia is a common laboratory abnormality in patients presenting with acute myocardial infarction (AMI) and always defined as a platelet count < 150 × 10^9^/L [4]. Baseline thrombocytopenia showed strong predictive capacity for major adverse clinical events (MACE), ischemic target lesion revascularization and 1-year death among patients with acute coronary syndrome (ACS) undergoing percutaneous coronary intervention (PCI) [5]. Additionally, thrombocytopenia has been previously considered as a risk factor for worse outcomes and has been reported to be independently associated with in-hospital mortality in ACS patients [6]. However, few studies have explored the prognostic value of thrombocytopenia for infection in patients with STEMI. In terms of etiological factors, in addition to congenital thrombocytopenia and pseudothrombocytopenia, different acquired causes can be included. Of which, infectious agents play a crucial role for their contribution to a reduction in the platelet count by suppressing the bone marrow directly or increasing peripheral consumption of platelets [7–9].

Recent studies have reported that patients with coronavirus disease 2019 (COVID-19) exhibited severe thrombocytopenia [10], and *Helicobacter pylori* (*H. pylori*) infection was on the rise as a cause of immune thrombocytopenia (ITP) [11], while another research demonstrated that low platelet count was independently correlated with an increased risk of infection in patients with ITP [12], which indicated that infections were closely associated with thrombocytopenia. Furthermore, thrombocytopenia has been shown to be a poor prognostic risk factor for systemic infections among patients from medical, surgical, mixed, or trauma ICUs [13], and moderate thrombocytopenia (platelet count < 100 × 10^9^/L) was a potential risk stratification tool for severe *Clostridium difficile* infection (CDI) [14].

Given the potential value of thrombocytopenia in the prediction of infection, and in order to improve risk stratification for these patients, we sought to investigate the association between thrombocytopenia and infection in patients with STEMI.

## Methods

### Study population

Patients diagnosed with STEMI were enrolled from January 2010 to June 2016 in Guangdong Provincial People’s Hospital. STEMI was diagnosed if patients presented within 12 h from the onset of symptoms including typical chest pain lasting for ≥ 30 min, not responsive to nitrates, with ST-segment elevation of ≥ 0.2 mV in at least two contiguous leads, or left bundle-branch block [15]. The exclusion criteria were as follows: (a) with chronic inflammatory disease; (b) with chronic renal failure necessitating hemodialysis upon hospital admission; (c) undergoing cardiac surgery; (d) who died within 24 h after hospital admission; (e) without performing PCI; (f) without platelet count data and (g) receiving dual antiplatelet therapy (DAPT) or other medications potentially causing thrombocytopenia before admission. The study protocol was approved by Ethics Committee of our hospital. The study was carried out according to the Principles of the Declaration of Helsinki 1975 and its later amendments. Written informed consents were obtained from patients or their relatives.

### Study endpoints and follow-up

Thrombocytopenia was defined as a platelet count < 150 × 10^9^/L [4]. The primary endpoint was the development of infection during hospitalization, which was defined as infection requiring antibiotics (reflecting the clinical influence of infection compatible with the necessity for additional treatment) [16], and was determined in medical records in accordance with ICD-10-CM codes, including pneumonia (J18.9), pyelonephritis (N10), ureteritis (N28.86), urethritis (N34.1), cystitis (N30.90), and other infections (such as sepsis, A41.9; bacteremia, R78.81 or unspecified infectious disease, B99.9). Additionally, the types of infections included pulmonary, urinary, or other (including abdominal sepsis, primary bacteremia, and unidentified primary infection site) infections. The secondary endpoint was in-hospital MACE, including all-cause death, stroke, and any bleeding during hospitalization. Other endpoints were also documented, including all-cause death during hospitalization and follow-up. Patients were followed up through telephone-tracking methods or outpatient clinic interviews for ≥ 1 year by trained nurses.

### Statistical analyses

Continuous variables with a normal distribution were presented as mean ± SD, and compared using two-sample *t*-tests. Continuous variables with a non-normal distribution were expressed as median and interquartile ranges, and analyzed using Wilcoxon rank sum tests. Categorical variables (which are presented as percentages) were compared using the chi-squared test or Fisher’s exact test (as appropriate). In-hospital outcomes and follow-up outcomes were calculated using a logistic regression model or Cox regression model in multivariable analysis. As for sample size consideration, we applied a rule of thumb, namely, that the number of events per variable should be 10 or greater. We expected about no more than 11 factors in the development model; thus, at least 110 infections were needed. According to the incidence of infections (10%) in the previous study [17], the final sample size of at least 1100 patients was needed. All tests were two-tailed, and *p* < 0.05 was considered significant. Data analyses were carried out using SAS v9.4 (SAS Institute, Cary, NC, USA).

## Results

**Fig. 1:**
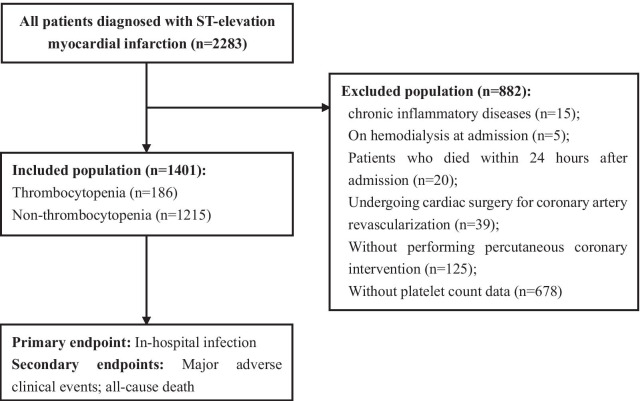
Flowchart showing patient selection

A total of 2283 patients with STEMI at our hospital were identified between January 2010 and June 2016; of these, 882 patients were excluded according to the exclusion criteria. Finally, 1401 patients (61.77 ± 12.37 years, 82.7% male) diagnosed with STEMI undergoing PCI were enrolled and analyzed in this study (Fig. 1). During hospitalization, 186 (13.3%) STEMI patients developed thrombocytopenia (platelet count < 150 × 10^9^/L). The clinical characteristics of the study population at baseline are shown in Table 1. Patients with thrombocytopenia were older and more likely to be men, than those not suffering from thrombocytopenia. A higher serum level of creatinine, and lower levels of total cholesterol and hemoglobin, were found in thrombocytopenic patients. Furthermore, thrombocytopenic patients had a higher prevalence of myocardial infarction and new use of antibiotics compared with non-thrombocytopenia cases. However, left ventricular ejection fraction (LVEF), procedural characteristics, and the prevalence of hypertension, diabetes mellitus, chronic obstructive pulmonary disease (COPD), and hyperlipemia were not significantly different between the two groups.

**Table 1:** Clinical characteristics of the study cohort

Variables	Overall (n = 1401)	Patients with thrombocytopenia (n = 186)	Patients without thrombocytopenia (n = 1215)	*p* value
Platelet counts (× 10^9^/L)	214.47 ± 64.55	125.81 ± 23.01	228.04 ± 57.76	< 0.001
Age (years)	61.77 ± 12.37	65.99 ± 11.06	61.13 ± 12.43	< 0.001
Male, n (%)	1158 (82.7%)	157 (84.4%)	1001 (82.4%)	0.498
Female, n (%)	243 (17.3%)	29 (15.6%)	214 (17.6%)	
SBP (mmHg)	122.00 ± 21.64	118.70 ± 21.82	122.50 ± 21.58	0.026
DBP (mmHg)	73.48 ± 12.76	71.63 ± 11.26	73.77 ± 12.96	0.034
Heart rate (bpm)	80.71 ± 16.54	80.78 ± 18.98	80.70 ± 16.14	0.954
Killip classification	1.42 ± 0.77	1.48 ± 0.88	1.41 ± 0.75	0.236
Hypertension, n (%)	724 (51.7%)	97 (52.2%)	627 (51.6%)	0.890
Diabetes, n (%)	367 (26.2%)	54 (29.0%)	313 (25.8%)	0.345
Hyperlipemia, n (%)	145 (10.3%)	14 (7.5%)	131 (10.8%)	0.175
Smoke, n (%)	622 (44.4%)	83 (44.6%)	549 (44.4%)	0.947
COPD, n (%)	34 (2.4%)	6 (3.2%)	28 (2.3%)	0.447
Prior MI, n (%)	72 (5.1%)	16 (8.6%)	56 (4.6%)	0.022
Prior stroke, n (%)	96 (6.9%)	19 (10.2%)	77 (6.3%)	0.051
Bleeding, n (%)	221 (15.8%)	46 (24.7%)	175 (14.4%)	< 0.001
Atrial fibrillation, n (%)	44 (3.1%)	76 (3.8%)	37 (3.0%)	0.601
CABG, n (%)	3 (0.2%)	0 (0.0%)	3 (0.2%)	0.498
Prior PCI, n (%)	140 (10.0%)	16 (8.6%)	124 (10.2%)	0.497
Laboratory characteristics				
White blood cell (× 10^9^/L)	11.82 ± 3.83	10.93 ± 3.52	11.95 ± 3.86	< 0.001
Serum creatinine (μmol/L)	103.78 ± 74.70	124.43 ± 104.83	100.61 ± 68.44	< 0.001
Triglycerides (mmol/L)	1.57 ± 1.20	1.35 ± 1.55	1.60 ± 1.14	0.010
Total cholesterol (mmol/L)	4.83 ± 1.19	4.33 ± 1.06	4.91 ± 1.19	< 0.001
LDL-C (mmol/L)	3.07 ± 1.01	2.65 ± 0.86	3.14 ± 1.02	< 0.001
HbA1c (%)	6.70 ± 1.73	6.85 ± 1.70	6.68 ± 1.74	0.271
Serum albumin (g/L)	33.26 ± 4.29	32.41 ± 4.11	33.39 ± 4.31	0.004
Hemoglobin (g/L)	136.24 ± 18.31	132.03 ± 19.59	136.88 ± 18.03	< 0.001
LVEF (%)	52.00 ± 11.71	50.79 ± 12.24	52.19 ± 11.61	0.130
Medication during hospital stay				
Statins, n (%)	1384 (98.8%)	178 (95.7%)	1206 (99.3%)	< 0.001
Aspirin, n (%)	1370 (97.8%)	181 (97.3%)	1189 (97.9%)	0.636
Clopidogrel, n (%)	1384 (98.8%)	178 (95.7%)	1206 (99.3%)	0.593
CCB, n (%)	149 (10.6%)	27 (14.5%)	122 (10.0%)	0.065
Olmesartan, n (%)	225 (16.1%)	30 (16.1%)	195 (16.0%)	0.978
New antibiotic use, n (%)	240 (17.1%)	53 (28.5%)	187 (15.4%)	< 0.001
Procedural characteristics				
Radial access, n (%)	1191 (85.0%)	155 (83.3%)	1036 (85.3%)	0.491
Femoral assess, n (%)	210 (15.0%)	31 (16.7%)	179 (14.7%)	
Contrast volume, (mL)	114.01 ± 42.81	117.86 ± 42.40	113.41 ± 42.86	0.213
Number of stents, (n)	1.30 ± 0.82	1.38 ± 0.88	1.28 ± 0.81	0.153
Total length of stent, (mm)	32.51 ± 22.38	34.67 ± 23.04	32.28 ± 22.27	0.158

Data are presented as mean ± SD or number (percentage)

*SBP* systolic blood pressure, *DBP* diastolic blood pressure, *COPD* chronic obstructive pulmonary disease, *MI* myocardial infarction, *TC* total cholesterol, *CABG* coronary artery bypass grafting, *PCI* percutaneous coronary intervention, *LDL-C* low-density lipoprotein cholesterol, *LVEF* left ventricular ejection fraction, *CCB* calcium channel blockers

As shown in Table 2, thrombocytopenic patients were more likely to develop infection (30.6% (57/186) vs. 16.2% (197/1215), *p* < 0.001), with no significant differences in various types of infection (*p* = 0.804), as well as in-hospital MACE (30.1% (56/186) vs. 16.4% (199/1215), *p* < 0.001) than non-thrombocytopenia cases during hospitalization. The thrombocytopenia group also had a higher proportion of in-hospital death (8.1% (15/186) vs. 3.8% (46/1215), *p* = 0.008) and longer hospital stay (11.41 ± 10.17 vs. 8.43 ± 6.80 days, *p* < 0.001) than the non-thrombocytopenia group (Table 2). Additionally, subgroup analysis revealed that 50 ≤ PLT < 150 was prominently associated with an increased risk of infection (odds ratio (OR), 2.21; 95% confidence interval (CI) 1.55–3.12; *p* < 0.001), but not severe thrombocytopenia defined as PLT < 50 (OR, 5.02; 95% CI 0.20–127.28; *p* = 0.255) due to the small sample size possibly (Additional file 1: Table S1). Conversely, an obvious negative correlation between normal platelet count and infection was discovered (OR, 0.20; 95% CI 0.17–0.23; *p* < 0.001) (Additional file 1: Table S1).

**Table 2:** In-hospital adverse events of the study cohort

Variables	Overall (n = 1401)	Patients with thrombocytopenia (n = 186)	Patients without thrombocytopenia (n = 1215)	*p* value
In-hospital infection, n (%)	254 (18.1%)	57 (30.6%)	197 (16.2%)	< 0.001
Type of infections, n (%)				0.804
Pulmonary infection	199 (78.3%)	43 (75.4%)	156 (79.2%)	
Urinary infection	33 (13.0%)	8 (14.0%)	25 (12.7%)	
Other infections	22 (8.7%)	6 (10.5%)	16 (8.1%)	
MACE, n (%)	255 (18.2%)	56 (30.1%)	199 (16.4%)	< 0.001
In-hospital death, n (%)	61 (4.4%)	15 (8.1%)	46 (3.8%)	0.008
Hospital day (d)	8.82 ± 7.40	11.41 ± 10.17	8.43 ± 6.80	< 0.001

Data are presented as mean ± SD or number (percentage)

*MACE* major adverse clinical events

Univariate analysis suggested that thrombocytopenia was significantly associated with an elevated risk of infection (OR, 2.28; 95%CI 1.61–3.22; *p* < 0.001). After adjustment for potential confounding factors (including age, Killip classification, diabetes, MI, stroke, serum albumin, WBC, hemoglobin, serum creatinine and LVEF), multivariate analysis showed that thrombocytopenia remained a significant predictor of infection risk in such patients (OR, 2.09; 95%CI 1.32–3.27; *p* = 0.001) (Table 3). Similar results were observed for the prevalence of in-hospital MACE (OR, 1.92; 95%CI 1.27–2.87; *p* = 0.002) but not all-cause death (OR, 1.87; 95%CI 0.88–3.78; *p* = 0.091) (Table 3).

**Table 3:** Adjusted OR and 95% CI of thrombocytopenia for infection, MACE and all-cause death at multivariate analysis

Variables	Thrombocytopenia		
	OR	95%CI	*p* value
Infection	2.09	1.32–3.27	0.001
MACE	1.92	1.27–2.87	0.002
All-cause death	1.87	0.88–3.78	0.091

Adjusted for age, Killip classification, diabetes, myocardial infarction, stroke, serum albumin, white blood cell, hemoglobin, serum creatinine and left ventricular ejection fraction

*OR* odds ratio, *CI* confidence interval, *MACE* major adverse clinical events

After a median follow-up of 2.85 years, univariate Cox proportional hazard regression analyses showed that thrombocytopenia was independently associated with increased all-cause death (hazard ratio (HR), 1.52; 95%CI 1.04–2.20; *p* = 0.029). However, upon fully adjusted multivariate Cox regression analysis, this significant association disappeared (adjusted HR, 1.19; 95%CI 0.80–1.77; *p* = 0.383) (Table 4).

**Table 4:** Cox proportional hazards regression analysis of factors associated with all-cause death

Variables	HR	95%CI	*p* value
Thrombocytopenia	1.19	0.80–1.77	0.383
Age	2.54	1.80–3.59	< 0.001
Killip classification	1.51	1.29–1.77	< 0.001
Diabetes	1.22	0.88–1.68	0.235
Myocardial infarction	1.57	0.93–2.66	0.094
Stroke	1.76	1.13–2.75	0.013
Serum albumin	0.73	0.48–1.10	0.128
White blood cell	1.56	1.10–2.20	0.013
Hemoglobin	0.10	0.99–1.04	0.311
Serum creatinine	1.02	1.01–1.04	< 0.001
LVEF	0.39	0.28–0.55	< 0.001

*HR* hazard ratio, *CI* confidence interval, *LEVF* left ventricular ejection fraction

## Discussion

The present study investigated, for the first time, the relationship between thrombocytopenia and infection in patients with STEMI undergoing PCI. We found that thrombocytopenia was associated with a significantly increased risk of in-hospital infection, in-hospital MACE but not all-cause death. These findings suggested that serial monitoring of platelet counts was essential to identify patients with STEMI at high risk of adverse outcomes.

Platelets are anucleate blood cells that play a crucial role in the maintenance of hemostasis [18]. Thrombocytopenia has been evaluated in various cardiovascular procedures [19, 20], and been proven to be associated with worse short-term and long-term outcomes in patients with ACS [5]. Williamson and colleagues reported that critically ill patients with thrombocytopenia had an increased risk of death compared with patients with a normal platelet count [21]. An increased risk of death, ischemic events, and bleeding has also been reported in thrombocytopenic patients with ACS without ST elevation upon electrocardiography [22]. In patients suffering from sepsis, thrombocytopenia reflected proinflammatory stimuli and pro-hemostatic mechanisms [23], which suggested that the more the pronounced infectious stimulus, the greater the risk of thrombocytopenia. Additionally, as reported previously, acute and chronic infectious diseases were common causes of thrombocytopenia [24]. However, the prognostic value of thrombocytopenia for infection remains unclear.

In our analyses, we demonstrated that the prevalence of thrombocytopenia in STEMI patients was 13.3%, while previous studies have reported the incidence of thrombocytopenia in patients with ACS varied from 1.6 to 13% [25, 26]. The mechanisms of platelet loss in such patients have not been illustrated explicitly because patients with thrombocytopenia are frequently excluded from large clinical trials [27]. However, anti-platelet agents and anticoagulation, especially heparin, are employed after surgical as well as conservative treatment, and may cause heparin-induced thrombocytopenia when antibodies react with complexes of heparin molecules and platelet factor-4 [28]. Furthermore, scholars have reported ischemic necrosis in patients presenting with myocardial dysfunction, was able to facilitate inflammation at the cellular level, and subsequently increased platelet clearance by macrophages as well as became the dominant stimulus for thrombocytopenia [26].

With regard to the predictive value of thrombocytopenia for infection and the underlying mechanisms of the association between them, some factors might be notable. First, patients with thrombocytopenia receive many transfusions of red blood cells and platelets in necessity, both of these interventions are proinflammatory [29, 30]. Second, infectious agents, with their prominent role in the causes of thrombocytopenia, help to decrease the platelet number by suppressing bone marrow directly or increasing peripheral consumption of platelets [8, 9]. Thus, to some extent, thrombocytopenia is able to reflect infection indirectly. Third, studies have demonstrated that platelets have a crucial role in response to infection by promoting translocation of immune cells to inflamed areas and releasing cytokines and other molecular mediators. Therefore, thrombocytopenia might inhibit platelet effects against infection by reducing the number of platelets and their function, which might be conducive to increasing the prevalence of in-hospital infection [31, 32]. Furthermore, platelets could inhibit macrophage-dependent inflammation, or enhance interleukin-10 secretion and reduce tumor necrosis factor-α (TNF-α) secretion during infectious and noninfectious systemic inflammation [33, 34]. Finally, in gram-negative pneumonia–derived sepsis in mice, thrombocytopenia was associated with a strongly impaired host defense via enhancing proinflammatory cytokine release and bacterial growth, and disrupting vascular integrity and activating endothelial cell [35].

In addition to thrombocytopenia, our study firstly found that the levels of serum albumin and hemoglobin was independently associated with the risk of infection. Leonardi and colleagues reported that declining hemoglobin content, with or without overt bleeding, was independently associated with heightened risk of 1-year mortality among patients with ACS managed invasively [36]. Plakht et al. demonstrated that decreased serum albumin level on admission was an independent prognostic marker of increased long-term all-cause mortality in patients with AMI [37], while hypoalbuminemia was also found to be significantly associated with worse in-hospital outcomes in patients with ACS [38]. However, we did not find that the levels of serum albumin and hemoglobin were associated with the risk of all-cause death, which might be attributed to the little sample size and low incidence of all-cause death in our study.

Our study highlights the notion that thrombocytopenia is infrequent in patients diagnosed with STEMI, but is associated with a markedly increased prevalence of infection and in-hospital MACE, and should not be underestimated. Early identification of thrombocytopenia is essential for appropriate clinical care of STEMI patients. Hence, dynamic monitoring of platelet count might be encouraged for cardiologists among high-risk patients to avoid complications.

This study also had several main limitations. First, the causes of thrombocytopenia were not elucidated clearly, and the diagnosis of thrombocytopenia according to one complete blood count (CBC) test might be not sufficient while minor variation might occur and platelet agglutination might result in technical CBC diagnosis of thrombocytopenia. Second, our study was carried out at a single center with a relatively small study cohort. Third, we evaluated a cohort of Chinese patients, so caution is needed when trying to apply our conclusions to another population. Fourth, we failed to collect the information about blood product transfusion, which might be a potential confounding factor. Finally, because of a lack of studies investigating the outcome of infection in thrombocytopenia patients, the potential molecular mechanisms could not be explored.

## Conclusions

In summary, the present study indicated that thrombocytopenia during hospitalization was independently associated with an increased prevalence of infection and in-hospital MACE in patients with STEMI. Thrombocytopenia could be used as a simple prognostic tool for these patients.

## Supplementary Information

**Additional file 1: Table S1.** Association of platelet count on infection after multivariable adjustment.

## Data Availability

The datasets used and/or analyzed in this study are available from the corresponding author on reasonable request.

## Abbreviations

STEMI: ST-elevation myocardial infarction
AMI: Acute myocardial infarction
ACS: Acute coronary syndrome
PCI: Percutaneous coronary intervention
MACE: Major adverse clinical events
ITP: Immune thrombocytopenia
DAPT: Dual antiplatelet therapy
TNF-α: Tumor necrosis factor-α
SBP: Systolic blood pressure
DBP: Diastolic blood pressure
COPD: Chronic obstructive pulmonary disease
TC: Total cholesterol
CABG: Coronary artery bypass grafting
LDL-C: Low-density lipoprotein cholesterol
LVEF: Left ventricular ejection fraction
CCB: Calcium channel blockers
CBC: Complete blood count
