# Supplementary material for: Association of thrombocytopenia and infection in patients with ST-elevation myocardial infarction undergoing percutaneous coronary intervention
Source: BMC Cardiovasc Disord. 2021 Aug 21;21:404. doi: 10.1186/s12872-021-02210-3 (PMC8379583; doi:10.1186/s12872-021-02210-3)
Supplement: Supplementary file 1 — Additional file 1: Table S1. Association of platelet count on infection after multivariable adjustment. [file 12872_2021_2210_MOESM1_ESM.docx]

**Additional file 1: Table S1.** Association of platelet count on infection after multivariable adjustment.

| **Platelet count** | **Sample size (n)** | **OR** | **95% CI** | ***P*-value** |
| --- | --- | --- | --- | --- |
| PLT ≥ 150 (Reference) | 1228 | 0.20 | 0.17-0.23 | ＜0.001 |
| 50 ≤ PLT＜150 | 183 | 2.21 | 1.55-3.12 | ＜0.001 |
| PLT＜50 | 2 | 5.02 | 0.20-127.28 | 0.255 |

*PLT* platelet count*, OR* odds ratio, *CI* confidence interval.
